# Potential contribution of *Helicobacter pylori* proteins in the pathogenesis of type 1 gastric neuroendocrine tumor and urticaria. *In silico* approach

**DOI:** 10.1371/journal.pone.0281485

**Published:** 2023-04-25

**Authors:** Andrés Sánchez Caraballo, Yenny Guzmán, Jorge Sánchez, Marlon Munera, Elizabeth Garcia, Deyanira Gonzalez-Devia

**Affiliations:** 1 Medical Research Group (GINUMED), University Corporation Rafael Núñez, Cartagena, Colombia; 2 Group of Clinical and Experimental Allergy (GACE), “IPS Universitaria” Clinic, University of Antioquia, Medellín, Colombia; 3 Department of Health Services, University of Washington, Seattle, WA, United States of America; 4 Facultad de Medicina, Universidad de los Andes, Bogotá, Colombia; 5 Departamento de Alergología, Fundación Santa Fe de Bogotá, Bogotá, Colombia; 6 UNIMEQ ORL, Bogotá, Colombia; Liv Hospital Gaziantep, TURKEY

## Abstract

**Background:**

*Helicobacter pylori* has been linked to several diseases such as chronic urticaria, gastritis, and type 1 gastric neuroendocrine tumors (type 1 gNET). Although these diseases seem to have different mechanisms, their relationship with *H*. *pylori* suggests a common inflammatory pathway.

**Objective:**

To identify potential cross-reactive antigens between *H*. *pylori* and humans involved in chronic urticaria and type 1 gNET.

**Methods:**

Alignment was carried out among human proteins associated with urticaria (9 proteins), type 1 gNET (32 proteins), and *H*. *pylori* proteome. We performed pairwise alignment among the human and *H*. *pylori* antigens with PSI-BLAST. Modeling based on homology was done with the Swiss model server and epitope prediction with the Ellipro server. Epitopes were located on a 3D model using PYMOL software.

**Results:**

The highest conserved sequence was found between the human HSP 60 antigen and the *H*. *pylori* chaperonin GroEL with an identity of 54% and a cover of 92%, followed by the alpha and gamma enolases and two *H*. *pylori* phosphopyruvate hydratase, both with an identity and cover of 48% and 96%, respectively. The H/K ATPase (Chain A) showed high identity with two *H*. *pylori* proteins (35.21% with both P-type ATPase), but with low cover (only 6%). We observed eight linear and three discontinuous epitopes for human HSP 60 and three lineal and one discontinuous epitope for both alpha-enolase and gamma enolase, high conserved with *H*. *pylori* sequences.

**Conclusion:**

Some type 1 gNET antigens shared potential cross-reactive epitopes with *H*. *pylori* proteins, suggesting that molecular mimicry could be a mechanism that explains the relationship between the infection and this disease. Studies evaluating the functional impact of this relationship are needed.

## Introduction

*Helicobacter pylori* (*H*. *pylori*) is a bacteria that colonize gastric mucosa in humans and increase the risk of serious diseases such as gastrointestinal ulcers and some cancers [1]. Most of the case, *H*. *pylori* cause a minimal damage to the gastric system and the immune response es effective in the bacterial elimination. However, in individual with a genetic predisposition, these bacteria have the ability to form subpopulations capable of immunomodulating the immune response and favoring a more severe inflammatory process, favoring effective harvest of nutrients to feed the bacteria and suppressing the immune response, allowing it to persist in the gastric system [2, 3]. The role of *H*. *pylori* infection in the pathogenesis of several extra-gastric diseases has been also suggested [4, 5].

Different mechanisms explain the pathogenicity of *H*. *pylori* and the initiation of the inflammatory process with the release of cytokines and chemokines [6]. These molecules facilitate the alteration of endothelial morphology and the passage of different proteins of the microorganism to the general circulation. Therefore, the role of *H*. *pylori* in different systemic diseases has been studied, and different mechanisms proposed; the systematic inflammatory could induce an autoimmune response generated by molecular mimicry between the proteins of *H*. *pylori* and humans [4, 7]. Molecular mimicry allows different microorganisms to evade the human immune system due to the similarity of some human proteins and microorganisms. However, when lymphocytes recognize these antigens, autoantibodies and/or autoreactive T lymphocytes can be generated.

Pathological processes that lead to Autoimmune Gastritis (AG) and type 1 gastric Neuroendocrine Tumors (type 1 gNETs) are not understood but some evidence suggests at both are autoimmune diseases in which the gastric mucosa is damaged and the production of autoantibodies against the exposed intracytoplasmic proteins are spread. Autoimmune atrophic gastritis is frequently found in association with Hashimoto’s thyroiditis, Graves disease, type 1 diabetes mellitus, Addison’s disease, and chronic urticaria [8]. Infection by *H*. *pylori* is common in AG, with a prevalence of 33,3 to 57%, however, the contribution of H pylori to type 1 gNET oncogenesis has not been demonstrated [9–11]. In chronic urticaria, autoimmunity has been strongly associated with its causality and severity [12, 13]. As in GA and gNETs, several epidemiological studies show a possible relationship between *H*. *pylori* and urticaria [14, 15], however, the reason for this association has not been explored.

The repertory of components common between *H*. *pylori* and the host has been increasing. From the *H*. *pylori* proteome, it is possible to evaluate the identity of its proteins in humans. Our hypothesis was the following: Considering that during chronic inflammatory processes there is an increased expression of some hidden proteins, it is possible that autoantibodies are generated against these proteins. In this study, through *in silico* analysis, we evaluated this hypothesis by comparing the identity of *H*. *pylori* proteins with some proteins identified in urticaria inflammation and in type 1 gNET, two extra-gastrointestinal systemic diseases with growing evidence of their possible association with *H*. *pylori*.

## Material and methods

### Antigen analysis

After an exhaustive review of the literature, we selected proteins for *in silico* analysis involved in urticaria, autoimmune thyroiditis, AG and type 1 gNET. A total of thirty-two amino acid sequences of tumoral neuroendocrine and AG antigens and nine antigens related with urticaria, and thyroiditis were retrieved from Uniprot database (Table 1).

**Table 1:** Tumoral neuroendocrine antigens and proteins associated with urticaria used to compare with the *H*. *pylori* proteome.

Antigens	Expression	UniProt
**Urticaria associated proteins**		
**Eosinophil peroxidase**	Eosinophil	P11678
**Eosinophil cationic protein**	Eosinophil	P12724
**Thyroid peroxidase**	Thyroid gland*	P07202
**Thyroglobulin**	Thyroid gland	P01266
**Myeloperoxidase**	Polymorphonuclear leukocytes	P05164
**Lactoperoxidase**	mammary glands, salivary gland, bronchial submucosal glands	P22079
**Peroxidasin like protein**	Cardiac muscle*	A1KZ92
**IL-24**	Adrenal gland*	Q13007
**High affinity immunoglobulin epsilon receptor subunit alpha**	Skin (187 others tissues)	P12319
**Type 1 pNETs proteins**		
**Hsp60**	Stomach, Endothelium,	P10809
**H/K ATPase (Chain A)**	Parietal cells	P20648
**H/K ATPase (Chain B)**	Parietal cells	P51164
**Carbonic anhydrase (9)**	Pancreas	Q16790
**E3 Ligase**	Pancreas	Q8IWV7
HLADR B1*0405	Pancreas	O19504
**Galactoside 3(4) -L-fucosyltransferase (Lewis antigens)**	Stomach, Endothelium, Platelets, Neutrophils	P21217
**Galactoside 2-alpha-L-fucosyltransferase 2 (Lewis antigens)**	Stomach, Endothelium, Platelets, Neutrophils	Q10981
**Platelet glycoprotein Ib Alpha chain (GPI)**	Platelets	P07359
**Platelet glycoprotein Ib Beta chain (GPII)**	Platelets	P13224
**Synaptophysin**	Brain, anterior cingulate cortex (185 other tissues)	P08247
**Neurofilament light proteins**	dorsal root ganglion (216 other tissues)	P07196
**Neurofilament medium proteins**	dorsal root ganglion (178 other tissues)	P07197
**Neurofilament heavy proteins**	dorsal root ganglion (193 other tissues)	P12036
**Chromogranin (A)**	Pancreas	P10645
**Neuronespecific Gamma Enolase**	Cerebellum (209 other tissues)	P09104
**Neuronespecific Alpha Enolase**	Kidney (244 other tissues)	P06733
**Glycoprotein hormones alpha chain (hCG alpha)**	Placenta (111 others tissues)	P01215
**Choriogonadotropin subunit beta (hCG Beta)**	Placenta	P0DN86
**Pancreatic Polypeptide**	Pancreas	P01298
**Alpha-fetoprotein**	Embryo	P02771
**Serotonin receptor**	Cerebral cortex, amygdala, hippocampus, testis (95 other tissues)	P46098
**Gastrin**	Stomach	P01350
**Insulin**	Pancreas (beta)	P01308
**Glucagon**	Pancreas (Alpha)	P01275
**Somatostatin**	Pancreas (Delta), hypothalamus	P61278
**Vasoactive intestinal peptide**	Vermiform appendix (141 other tissues)	P01282
**Histamine 2 receptor**	Nerve cells, airway and vascular smooth muscles, endothelium, epithelium, leukocytes	P25021
**Histamine 3 receptor**	Nerve cells, airway and vascular smooth muscles, endothelium, epithelium, leukocytes	Q9Y5N1
**Histamine 4 receptor**	Nerve cells, airway and vascular smooth muscles, endothelium, epithelium, leukocytes	Q9H3N8
**Transient receptor potential ankyrin 1**	Oocyte, liposarcoma cells, fibroblasts	O75762
**Calcitonin**	Dorsal root ganglion (128 other tissues)	P01258
**Galanin**	Adenohypophysis (118 other tissues)	P22466

Amino acid sequences of each antigen was used as input in PSI-BLAST to find similar antigens from *H*. *pylori* and alignment using two algorithms, BLOSUM62 through PRALINE pairwise from the center for integrative bioinformatics IBIVU (https://www.ibi.vu.nl/programs/pralinewww/) and PAM250 using the EMBOSS Needle pairwise (https://www.ebi.ac.uk/Tools/psa/emboss_needle/), based on the assumption of both short and long evolutionary distances, respectively. For the progressive alignment strategy, gap penalty was used of 12 opens, 0.5 and 1 extension, with an iteration of 3. Search was limited to taxid 210, corresponding to the specific species *H*. *pylori*. Antigens with similarity lower than 30% were discard.

### Modelling based on homology

The models were used to locate residues exposed and conserved on the surface to conformation of antigenic patches. Antigens with experimental structure resolved were retrieved from Protein Data Bank. 3D structures from antigens not reported in Protein Data Bank were generated by modelling based on homology with Swiss Model server (https://swissmodel.expasy.org/interactive) and were refined with Deep View for energy minimization. Its quality was evaluated by several tools, including the Ramachandran graphs, WHATIF, QMEAN4 index and energy values (GROMOS96 force field). All models were visualized with Pymol 2.3 [16, 17].

### Epitope prediction

B cell epitope prediction was made with Ellipro server (http://tools.iedb.org/ellipro/). Prediction parameters were set up as default. Also, antigenic patches reported were retrieved to explore molecular mimicry between tumoral And *H*. *pylori* antigens. Only epitopes with a score above of 0,7 and more than 4 residues were selected [16, 17].

## Results

### *H*. *pylori* antigens selection

From the thirty-two tumoral neuroendocrine and AG human antigen sequences retrieved, eight share identities with ten *H*. *pylori* proteins: The Lewis antigens (3-galactosyl-N-acetylglucosaminide 4-alpha-L-fucosyltransferase and Galactoside alpha-(1,2)-fucosyltransferase 2), heat shock protein 60 (HSP 60), H/K ATPase, carbonic anhydrase, the heavy neurofilament protein and the neuro-specific alpha and gamma enolases. The H/K ATPase was the only one that present identity with more than one *H*. *pylori* protein (Table 2).

**Table 2:** Pairwise alignment results from human proteins and *H*. *pylori* proteins using Blosum 62 and PM250 algorithms.

Human Protein	H. pylori Protein (NCBI ID)	Identity BLOSUM 62 (%)	Identity PM250 (%)	Cover (%)
**Lewis antigens**				
Galactoside alpha-(1,2)-fucosyltransferase 2	Alpha-1,2-fucosyltransferase (WP_128060420.1)	25%	24,54%	69%
3-galactosyl-N-acetylglucosaminide 4-alpha-L-fucosyltransferase	fucosyltransferase (WP_100980580.1)	21%	25%	22%
**HSP60**	chaperonin GroEL (WP_140474878.1)	54%	53,76%	92%
**H/K ATPase**				
H/K ATPase (Chain A)	Copper-translocating P-type ATPase CopA (WP_127984181.1)	17%	22,97%	11%
	HAD-IC family P-type ATPase, partial (WP_164532520.1)	35,21%	28%	6%
	cadmium-translocating P-type ATPase (WP_096470154.1)	35,21%	30%	6%
H/K ATPase (Chain B)	No significant similarity found			
**Carbonic anhydrase**	Carbonic anhydrase (WP_139531711.1)	27%	26,89%	48%
**E3 ligase**	No significant similarity found.			
HLADR B1*0405	No significant similarity found.			
**GP I/II**	No significant similarity found.			
**Synaptophysin**	No significant similarity found.			
**Neurofilament proteins**				
Light	No significant similarity found.			
Medium	No significant similarity found.			
Heavy	Hypothetical protein (WP_000782088.1)	19%	25%	16%
**Chromogranin (A)**	No significant similarity found.			
**Neuronespecific Alpha Enolase**	phosphopyruvate hydratase (WP_120924717.1)	48%	47,38%	96%
**Neuronespecific Gamma Enolase.**	phosphopyruvate hydratase (WP_025275561.1)	48%	48,81%	96%
**HCG**				
Alpha	No significant similarity found.			
Beta	No significant similarity found.			
**Pancreatic Polypeptide**	No significant similarity found.			
**Alpha-fetoprotein**	No significant similarity found.			
**Serotonin**	No significant similarity found.			
**Gastrin.**	No significant similarity found.			
**Somatostatin**	No significant similarity found.			
**Vasoactive intestinal peptide**	No significant similarity found.			

The cover and identity values were taken to establish the degree of relationship between the proteins. The results are expressed as a percentage.

None of the nine proteins associated with urticaria and thyroiditis showed identity with the *H*. *pylori* proteome.

All the human proteins with significant identity, presented functional homology with the *H*. *pylori* proteins and have enzymatic functions: two fucosyltransferases, three ATPases, one carbonic anhydrase and two phosphopyruvate hydratase. There were not a high different in the identities between the BLOSUM62 and PAM250 algorithms. The highest conserved sequence was found in the HSP 60 antigen, with an identity of 54% and a cover of 92%, followed by the alpha and gamma enolases, both had an identity level of 48% and cover of 96%. The H/K ATPase showed high identity with two *H*. *pylori* proteins (35,21% with both HAD IC family P type ATPase and Cadmium-translocating P-type ATPase), but the cover were low (only 6%). The lowest conserved sequence was present with the third H/K ATPase pairwise, with an identity of 17% and cover of 11%.

### Modelling and epitope prediction

From the eight tumoral neuroendocrine human antigens, five did not present a 3D experimental structure: HSP60, H/K ATPase (Chain A), heavy neurofilament protein, and the two Lewis antigen (FUT2 and FUT3) and were modelling based on homology with the Swiss Model server. The templated used were 60 kDa heat shock protein mitochondrial homology (100%) for HSP60, Potassium-transporting ATPase alpha chain 1 (96,71%) for H/K ATPase, Keratin type I cytoskeletal 10 (37,5%) for the heavy neurofilament protein and Alpha-(1,6)-fucosyltransferase (17,27%) and Alpha1,3-fucosyltransferase (21,95%) for the two Lewis antigens, FUT 2 and FUT 3, respectively. Models showed typical fold expected for their protein family. For those whose did not fit in range of 0–1 was refined in Deep view software.

By using Ellipro server, the lineal and discontinuous epitopes on the tumoral neuroendocrine protein were predicted. By the selection criteria, the server threw four lineal and four discontinuous for FUT 2, three both lineal and discontinuous epitopes for FUT 3, eight lineal and three discontinuous epitopes for HSP 60, three lineal epitopes for carbonic anhydrase and non-valid discontinuous epitopes, three lineal and one discontinuous epitopes for alpha enolase and gamma enolase, nine lineal and three discontinuous epitopes for H/K ATPase and none lineal but three discontinuous epitopes for the heavy neurofilament protein. For each of these proteins, some epitopes presented a possible antigenic patch among the alignment, in sequence with a high homology as of see in Table 3.

**Table 3:** Lineal and discontinuous epitopes from human HSP60, alpha enolase and gamma enolase, predicted by Ellipro server.

Epitope	Start	End	Peptide	Number of residues	Score
HSP60					
LE 1	538	552	LLTTAEVVVTEIPKE	15	0.853
LE 2	276	299	EDVDGEALSTLVLNRLKVGLQVVA	24	0.794
LE 3	127	143	GFEKISKGANPVEIRRG	17	0.756
LE 4	321	347	GGAVFGEEGLTLNLEDVQPHDLGKVGE	27	0.743
LE 5	446	466	RCIPALDSLTPANEDQKIGIE	21	0.739
LE 6	84	93	IDLKDKYKNI	10	0.728
DE 1			I:A27, I:K28, I:D29, I:V30, I:K31, I:F32, I:G33, I:A34, I:D35, I:A36, I:R37, I:A38, I:L39, I:M40, I:L41, I:Q42, I:I84, I:D85, I:L86, I:D88, I:K89, I:Y90, I:K91, I:N92, I:I93, I:G127, I:F128, I:E129, I:K130, I:I131, I:S132, I:K133, I:G134, I:A135, I:N136, I:P137, I:V138, I:E139, I:I140, I:R141, I:R142, I:G143, I:L146, I:D149, I:A150, I:A153, I:E154, I:K157, I:Q158, I:S159, I:K160, I:P161, I:V162, I:T163, I:T164, I:P165, I:E166, I:E167, I:G435, I:C447, I:I448, I:P449, I:A450, I:L451, I:D452, I:S453, I:L454, I:T455, I:P456, I:A457, I:N458, I:E459, I:D460, I:Q461, I:K462, I:I463, I:G464, I:I465, I:E466, I:K469, I:L538, I:L539, I:T540, I:T541, I:A542, I:E543, I:V544, I:V545, I:V546, I:T547, I:E548, I:I549, I:P550, I:K551, I:E552	95	0.747
DE 2			I:G222, I:Y223, I:I224, I:S225, I:P226, I:Y227, I:F228, I:I229, I:N230, I:T231, I:S232, I:K233, I:G234, I:Q235, I:K236, I:C237, I:E238, I:F239, I:Q240, I:D241, I:A242, I:Y243, I:L245, I:S247, I:E248, I:K249, I:K250, I:I251, I:S252, I:S253, I:I254, I:Q255, I:S256, I:I257, I:V258, I:P259, I:A260, I:L261, I:E262, I:I263, I:A264, I:N265, I:A266, I:H267, I:R268, I:K269, I:P270, I:L271, I:I273, I:E276, I:D277, I:V278, I:D279, I:G280, I:E281, I:A282, I:L283, I:S284, I:T285, I:L286, I:V287, I:L288, I:N289, I:R290, I:L291, I:K292, I:V293, I:G294, I:L295, I:Q296, I:V297, I:V298, I:A299, I:G321, I:G322, I:A323, I:V324, I:F325, I:G326, I:E327, I:E328, I:G329, I:L330, I:T331, I:L332, I:N333, I:L334, I:E335, I:D336, I:V337, I:Q338, I:P339, I:H340, I:D341, I:L342, I:G343, I:K344, I:G346, I:E347, I:T351, I:K352, I:L358, I:K359, I:G360, I:K361, I:G362, I:D363, I:K364, I:A365, I:Q366, I:K369	111	0.734
DE 3			I:E65, I:Q66, I:S67, I:W68, I:G69, I:S70, I:P71	7	0.731
Alpha enolase					
LE 1	49	105	RDNDKTRYMGKGVSKAVEHINKTIAPALVSKKLNVTEQEKIDKLMIEMDGTENKSKF	57	0.729
LE 2	250	287	FFRSGKYDLDFKSPDDPSRYISPDQLADLYKSFIKDYP	38	0.726
LE 3	224	239	ELLKTAIGKAGYTDKV	16	0.706
DE 1			A:S1, A:I2, A:L3, A:K4, A:I5, A:H6, A:A7, A:R8, A:E9, A:F11, A:D12, A:G15, A:N16, A:D22, A:L23, A:F24, A:T25, A:S26, A:K27, A:G28, A:L29, A:F30, A:L46, A:L48, A:R49, A:D50, A:N51, A:D52, A:K53, A:T54, A:R55, A:Y56, A:M57, A:G58, A:K59, A:G60, A:V61, A:S62, A:K63, A:A64, A:V65, A:E66, A:H67, A:I68, A:N69, A:K70, A:T71, A:I72, A:A73, A:P74, A:A75, A:L76, A:V77, A:S78, A:K79, A:K80, A:L81, A:N82, A:V83, A:T84, A:E85, A:E87, A:K88, A:I89, A:K91, A:L92, A:I94, A:E95, A:M96, A:D97, A:G98, A:T99, A:E100, A:N101, A:K102, A:S103, A:K104, A:F105, A:A120, A:G121, A:A122, A:V123, A:E124, A:K125, A:G126, A:V127, A:P128	87	0.722
Gamma enolase					
LE 1	251	287	YRDGKYDLDFKSPTDPSRYITGDQLGALYQDFVRDYP	37	0.737
LE 2	47	105	ELRDGDKQRYLGKGVLKAVDHINSTIAPALISSGLSVVEQEKLDNLMLELDGTENKSKF	59	0.714
LE 3	224	239	ELVKEAIDKAGYTEKI	16	0.698
DE 1			A:S1, A:I2, A:E3, A:K4, A:I5, A:W6, A:A7, A:R8, A:E9, A:L11, A:D12, A:G15, A:N16, A:D22, A:L23, A:Y24, A:T25, A:A26, A:K27, A:G28, A:L29, A:F30, A:L46, A:L48, A:R49, A:D50, A:G51, A:D52, A:K53, A:Q54, A:R55, A:Y56, A:L57, A:G58, A:K59, A:G60, A:V61, A:L62, A:K63, A:A64, A:V65, A:D66, A:H67, A:I68, A:N69, A:S70, A:T71, A:I72, A:A73, A:P74, A:A75, A:L76, A:I77, A:S78, A:S79, A:G80, A:L81, A:S82, A:V83, A:V84, A:E85, A:E87, A:K88, A:L89, A:N91, A:L92, A:L94, A:E95, A:L96, A:D97, A:G98, A:T99, A:E100, A:N101, A:K102, A:S103, A:K104, A:F105, A:A120, A:G121, A:A122, A:A123, A:E124, A:R125, A:E126, A:L127, A:P128, A:R131	88	0.722

LE: Lineal epitope. DE: discontinuous epitope.

**Fig 1:**
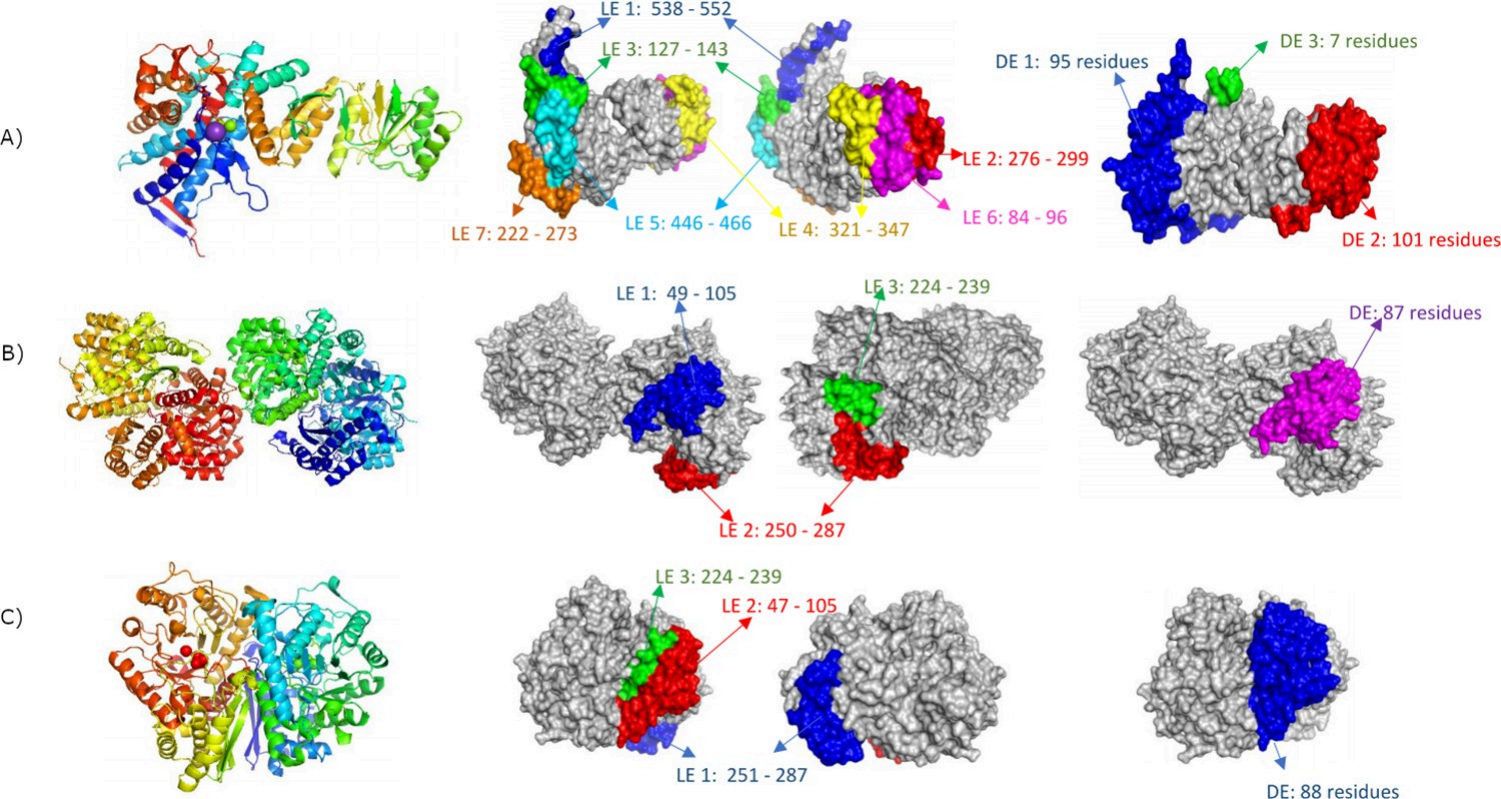
3D structure of the human HSP60 (A), alpha enolase (B) and gamma enolase (C). Cartoon and surface models are showed to represent the position of the epitope predicted in the human antigens. All the epitopes are indicated in color on surface models: blue, red, green, yellow, cyan, magenta, orange. LE. Lineal epitope. DE: discontinuous epitope.

**Fig 2:**
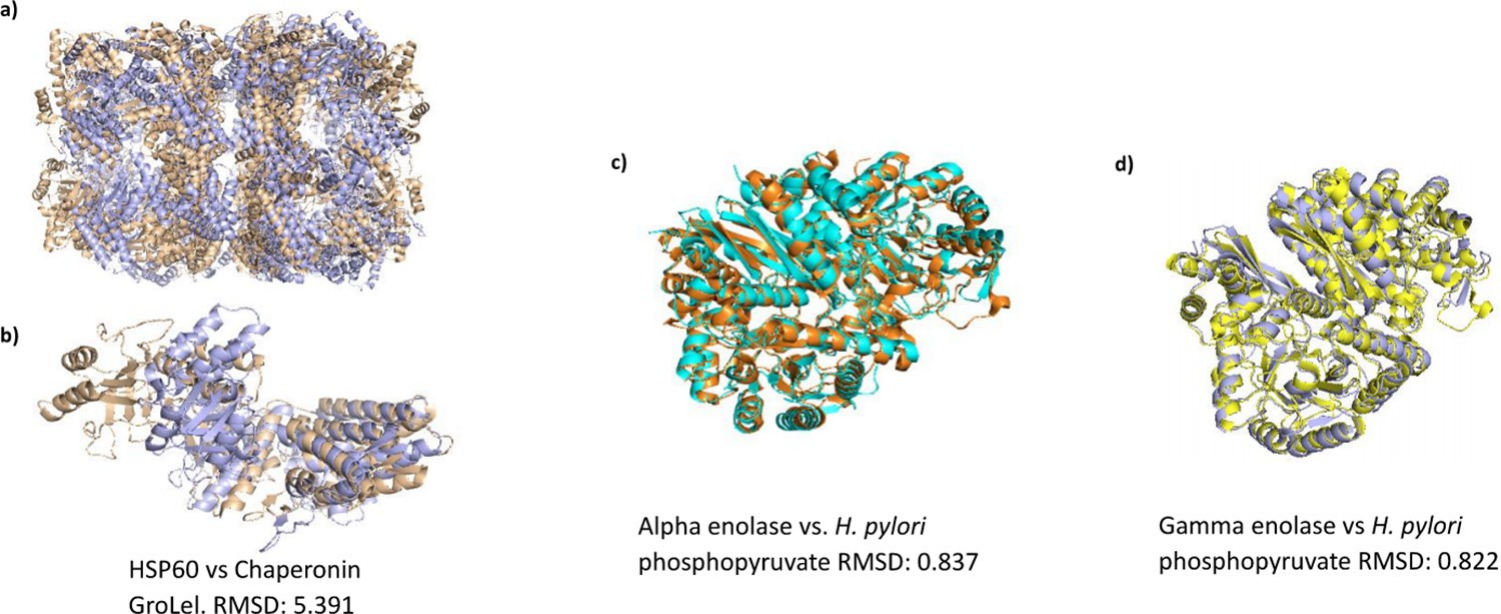
Overlay of the 3-D models between type 1 gNET antigens and *H*. *pylori* proteins. Samples: human HSP60 (gold) vs H. pylori chaperonin Groel (Magenta) are shown as the complete unit (**A**) and a subunit (**B**). (**C**) Human Alpha enolase (orange) vs *H*. *pylori* phosphopyruvate (cyan). (**D**) Gamma enolase (gray) vs phosphopyruvate (yellow). RMSD: Root-mean-square deviation are showed in Armstrong (Å).

In the case of HSP60, the second epitope (LE2: 276–299) was in the most conserved region compared to the other three epitopes. For the alpha enolase, the first epitope described (LE1: 49–106) was the largest one of the three antigenic patches, with a total of 57 residues and 26 (45,6%) of them being identical with the *H*. *pylori* protein. By last, gamma enolase presents one conservated epitope of 59 (LE2: 47–105), with an identity of 45,76% with the *H*. *pylori* protein (Fig 1). The 3D models of the human HSP60, alpha enolase and gamma enolase shown a similar arrangement with *H*. *pylori* proteins chaperonin GroEL and the two phosphopyruvate, respectively. The furthest RMSD was observed between HSP60 and chaperonin GroEL, with a value of 5.391, however, PDB validation of HSP60 are low and may have a low molecular spatial distribution. The overlay of the 3D models between the three human antigens and the *H*. *pylori* whit the highest identity values are shown in the Fig 2.

## Discussion

Despite the multiple epidemiological studies that associate infection with *H*. *pylori* and various systemic diseases, it is not clear if this association is involved in the pathogenesis of these diseases [6, 18–22]. One of the most discussed mechanisms is the induction of autoimmunity by *H*. *pylori* through molecular mimicry [23, 24], but few human antigenic proteins have been associated with this mechanism and they vary depending on the type of disease [1, 4, 25]. Also, studies are lacking to show that these autoantibodies are functional.

In urticaria, different proteins have been described that can be recognized by IgG, IgM or IgE autoantibodies [12, 26, 27], some of these autoantibodies can induce the activation of basophils and mast cells, indicating that they can induce an inflammatory response [12, 28]. Some studies suggest that the frequency of *H*. *pylori* infection is higher in patients with urticaria [29]; case series have been published where elimination of *H*. *pylori* has been associated with remission of the disease [15]. Although these results are still controversial [30, 31] suggest that the presence of *H*. *pylori* may contribute to the development of urticaria. When we analyzed the nine human antigen proteins that have been recognized by autoantibodies with functional activity, none shared identity with the *H*. *pylori* proteins analyzed in this study. Although our results suggest that there is no relationship between *H*. *pylori* and urticaria through molecular mimicry, they also do not completely rule out this hypothesis; Schmetzer et al. [27], observed that more than 200 human proteins could be recognized by IgE autoantibodies in patients with urticaria, therefore *H*. *pylori* proteins could have molecular mimicry with other proteins that we did not evaluate; nevertheless, not all those autoantibodies are functional, and the 9 proteins included in this study are those that have antibodies with functional studies. There are also proposals for other mechanisms that could explain the relationship between *H*. *pylori* and urticaria not dependent on molecular mimicry [32].

In the case of type 1 gNET, there is also controversy about the role of *H*. *pylori* [33, 34] but its association with other types of cancer, specially gastric cancer is strong [7, 22, 24]; Following our hypothesis of a possible cross-reactivity between *H*. *pylori* proteins and human proteins associated with type 1 gNET, we found that HSP60 from *H*. *pylori* and human share identity and we identified possible antigenic patches.

HSP60 is a mitochondrial localized quality control protein responsible for maintaining mitochondrial function. HSP60 is considered both a tumor suppressor and promoter in different types of cancer; its role in the oncogenesis of type 1 gastric neuroendocrine tumor needs to be explored. HSPs participate as immunomodulators in both innate and in both innate and acquired immune responses [35, 36].

Enolase is a dimeric protein composed of three isoenzymes, alpha, beta, and gamma (α, β and γ). This enzyme catalyzes the phosphoenolpyruvate and 2-phosphoglycerate, one of the last steps in glycolysis. Enolase, specially α enolase, have been widely used as markers of NETs through immunostaining. Glycolysis is a fundamental metabolic pathway, and its enzymes are highly conserved and present in procaryotic and eukaryotic species, including pathogens, like *Streptococcus aureus*, *Streptococcus pneumoniae*, *Candida albicans*, *and Leishmania mexicana*, make it an excellent candidate to produce cross-reactive autoantibodies.

These results show the utility of *in silico* assays for clinical research; without putting patients at risk, *in silico* analysis allows us to explore research hypotheses and prioritize resources in those that are most promising. At the same time, it allows the integration of multiple disciplines, which generates a more holistic vision in the approach and study of patients. According to these results, subsequent investigations of the role of *H*. *pylori* in urticaria require a different hypothesis, while in the case of NETs the results suggest that some autoantigens could explain the relationship between *H*. *pylori* in a subgroup of patients.

HSP60 and enolase have previously been identified in other gastrointestinal disorders like autoimmune colitis and Chron’s disease [37, 38]. Therefore, our results support those autoantibodies against the human homonymous protein may have some role in this disease, but it is necessary to carry out functional studies to evaluate whether these antigens, when recognized by the immune system, generate an inflammatory response. It is also necessary to note that our study has some weaknesses that could explain the lack of cross-reactivity between the urticaria auto-antigens, and the *H*. *pylori* antigens evaluated; Due to the lack of knowledge about the various proteins involved in urticaria and gNET, some important molecules could not be included, and this could explain the lack of association between the pathogen and urticaria. In addition, it is necessary to explore other mechanisms such as epitope spreding, which may also be associated with this relationship between urticaria, type 1 gNET and *H*. *pylori*.

However, these autoantibodies can be useful as biomarkers with clinical utility. Molecular mimicry usually occurs by evolutionary conservation of proteins between different species; with the results of our study, we identified for the first-time shared epitopes between *H*. *pylori* proteins and human proteins related to the pathogenesis of type 1 gNET. This finding may have biological implications such as the potential formation of an autoimmune response mediated by antibodies secondary to the recognition of *H*. *pylori* proteins. Previous analyses, comparing *in silico* techniques versus functional techniques [39, 40], show that in silico analyzes allow to detected proteins that share identity with a 90% of precision, therefore they are quite useful for the development of new research hypotheses and they are cost/effective since they allow a better administration of research resources, especially those where there is little information with functional studies, as is the case of our study where we explored the relationship between *H*. *pylori*, urticaria and type 1 gNET; our results provide a rational basis for future research between *H*. *pylori* and type 1 gNET but advise against such research in urticaria where other mechanisms or proteins should be evaluated.

In conclusion, some human proteins associated with type 1 gNET like the HSP60 and enolases retain common epitopes with *H*. *pylori* proteins, suggesting that molecular mimicry could be a mechanism that explains the relationship between the microorganism and this disease. Our results allowed us to identify possible epitopes with molecular mimicry between *H*. *pylori* and type 1 gNET; these regions are the most likely to be associated with cross-reactivity; however, cross-reactivity requires in vitro studies to confirm. Additionally, our results suggest with a high level of certainty that the probability of molecular mimicry between *H*. *pylori* and the evaluated human urticaria-related proteins is low, so it is unlikely that they present cross-reactivity, which suggests that new proteins should be investigated, or other mechanisms explored to determine the relationship between *H*. *pylori* and chronic urticaria. However, studies evaluating the functional impact of this relationship are necessary.

## Supporting information

S1 Fig Pairwise alignment between human FUT 2 and 1,2 fucosyltransferase. Unconserved sequence are shown with blue color and high conserved sequence with red color. Moderately conserve sequence is showed with green and orange color.
(TIFF)

S2 Fig Pairwise alignment between human FUT3 vs 1,3 fucosyltransferase. Unconserved sequence are shown with blue color and high conserved sequence with red color. Moderately conserve sequence is showed with green and orange color.
(TIFF)

S3 Fig Pairwise alignment between human HSP 60 vs chaperonin Gro EL. Unconserved sequence are shown with blue color and high conserved sequence with red color. Moderately conserve sequence is showed with green and orange color.
(TIFF)

S4 Fig Pairwise alignment between human carbonic anhydrase vs carbonic anhydrase. Unconserved sequence are shown with blue color and high conserved sequence with red color. Moderately conserve sequence is showed with green and orange color.
(TIFF)

S5 Fig Pairwise alignment between human Alpha enolase vs phosphopyruvate. Unconserved sequence are shown with blue color and high conserved sequence with red color. Moderately conserve sequence is showed with green and orange color.
(TIFF)

S6 Fig Pairwise alignment human gamma enolase vs phosphopiruvate hydratase. Unconserved sequence are shown with blue color and high conserved sequence with red color. Moderately conserve sequence is showed with green and orange color.
(TIFF)

S7 Fig Pairwise alignment between human H/K ATPase vs cooper translocate ATPase. Unconserved sequence are shown with blue color and high conserved sequence with red color. Moderately conserve sequence is showed with green and orange color.
(TIFF)
