# Supplementary figures and images for: Potential contribution of Helicobacter pylori proteins in the pathogenesis of type 1 gastric neuroendocrine tumor and urticaria. In silico approach
Source: PLoS One. 2023 Apr 25;18(4):e0281485. doi: 10.1371/journal.pone.0281485 (PMC10128923; doi:10.1371/journal.pone.0281485)

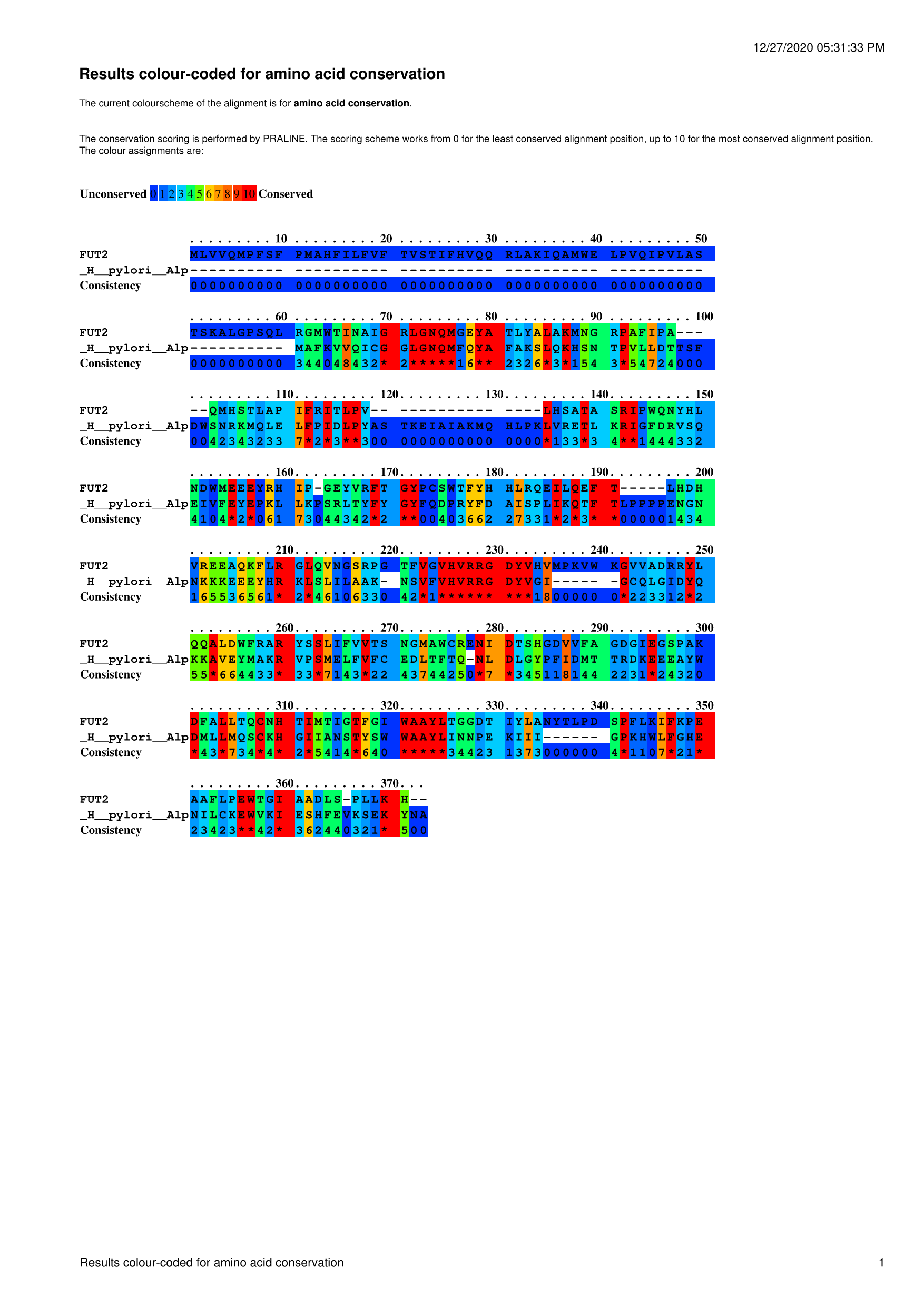

Supplement: S1 Fig — Unconserved sequence are shown with blue color and high conserved sequence with red color. Moderately conserve sequence is showed with green and orange color. (TIFF) [file pone.0281485.s001.tiff]

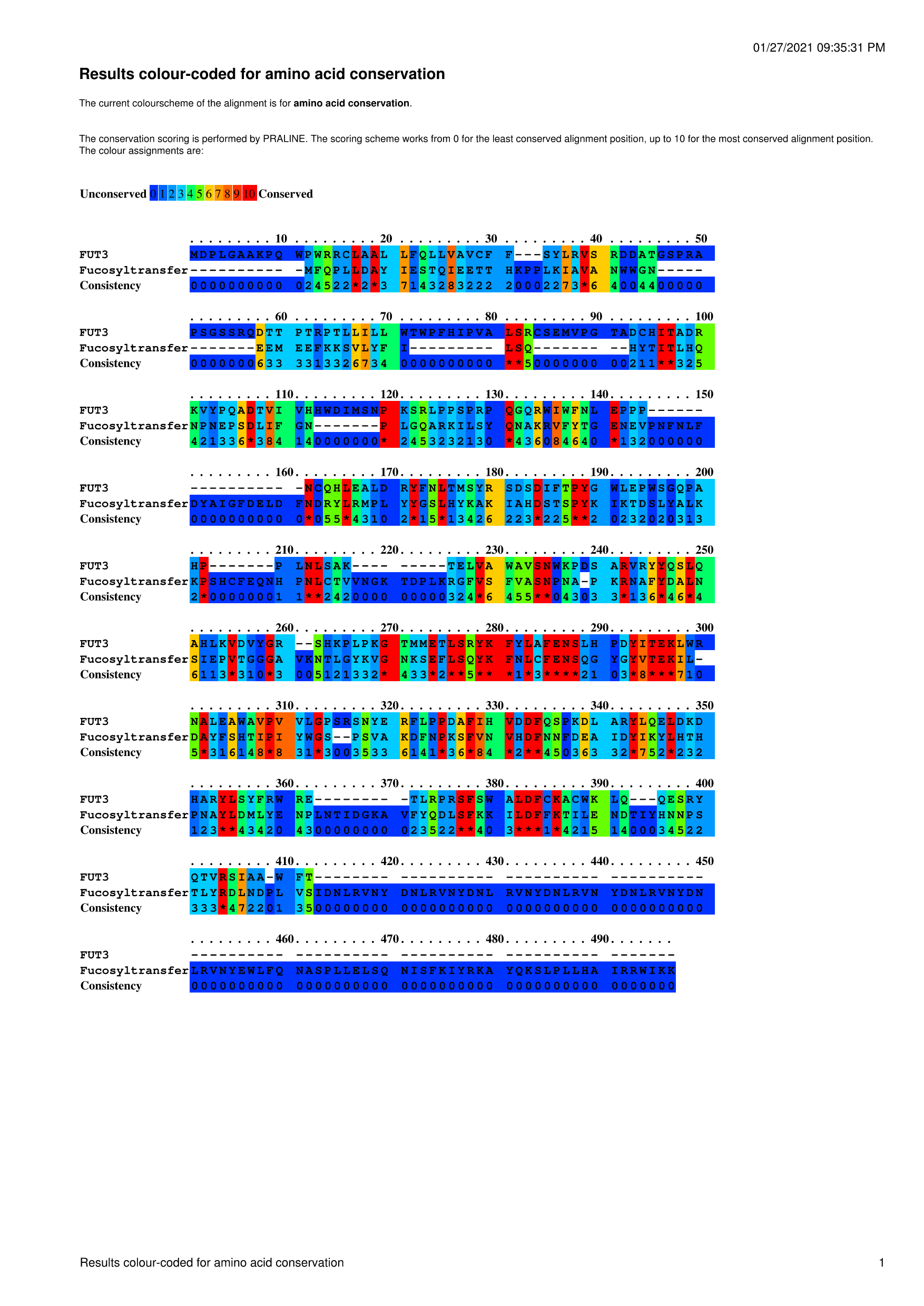

Supplement: S2 Fig — Unconserved sequence are shown with blue color and high conserved sequence with red color. Moderately conserve sequence is showed with green and orange color. (TIFF) [file pone.0281485.s002.tiff]

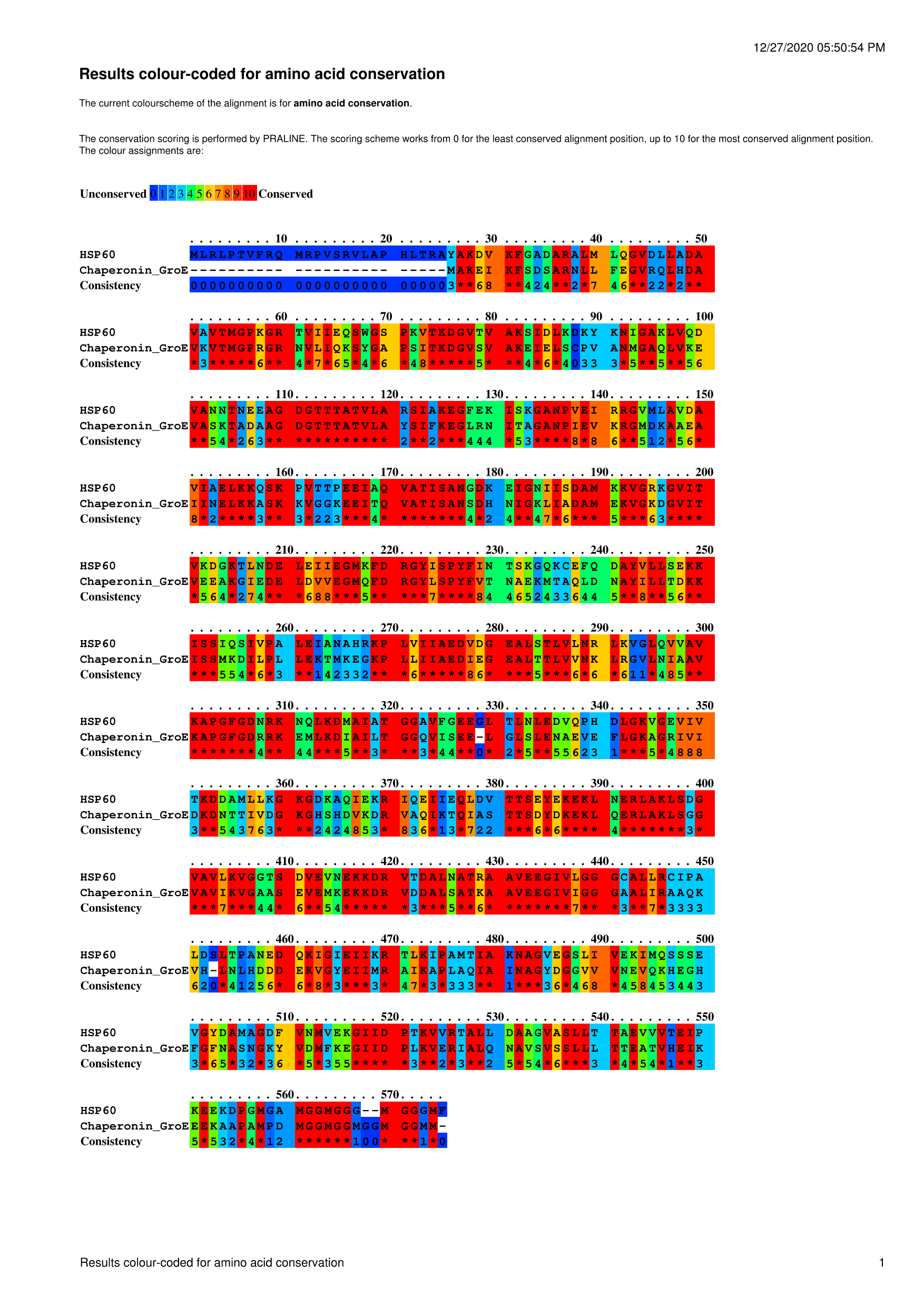

Supplement: S3 Fig — Unconserved sequence are shown with blue color and high conserved sequence with red color. Moderately conserve sequence is showed with green and orange color. (TIFF) [file pone.0281485.s003.tiff]

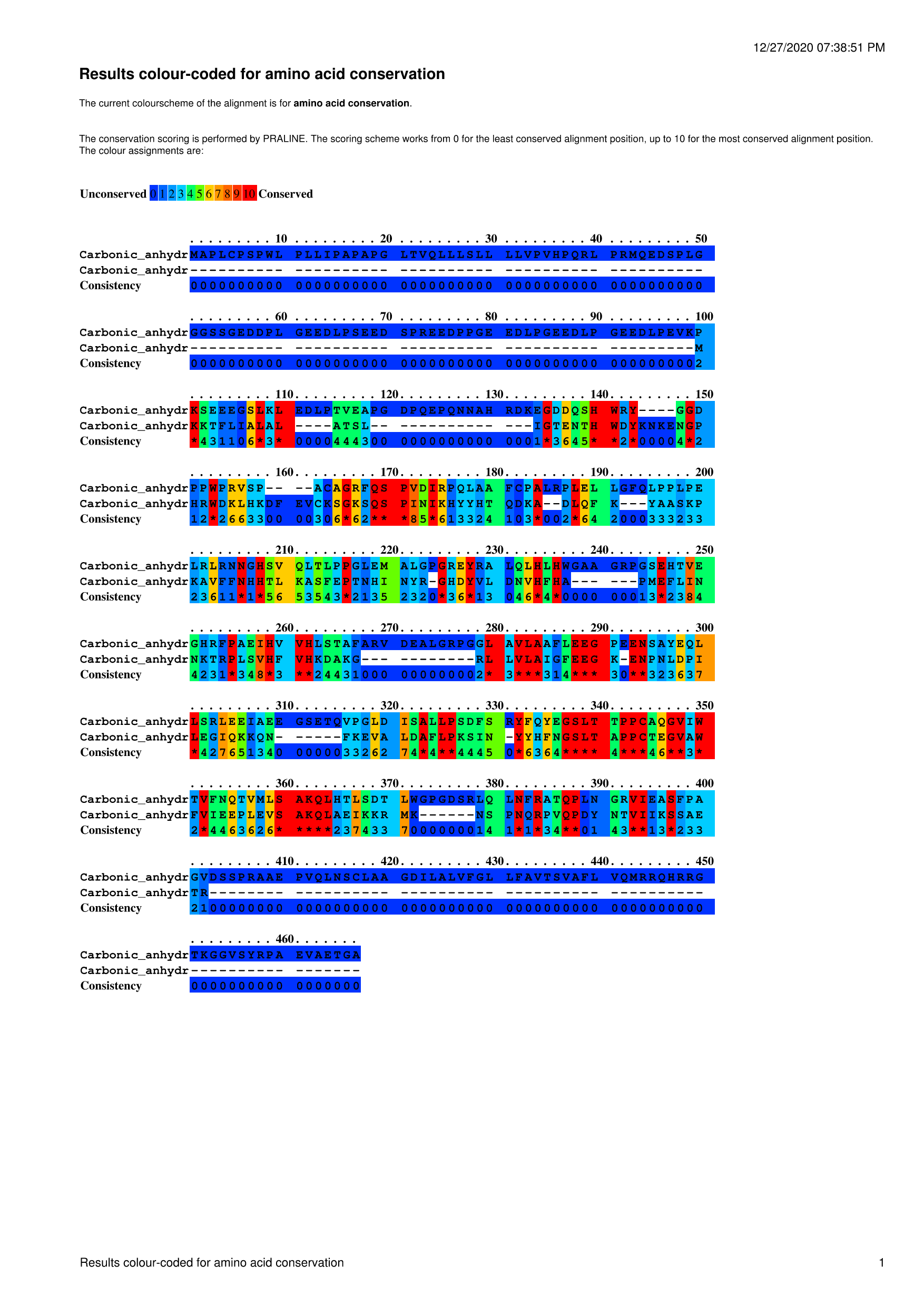

Supplement: S4 Fig — Unconserved sequence are shown with blue color and high conserved sequence with red color. Moderately conserve sequence is showed with green and orange color. (TIFF) [file pone.0281485.s004.tiff]

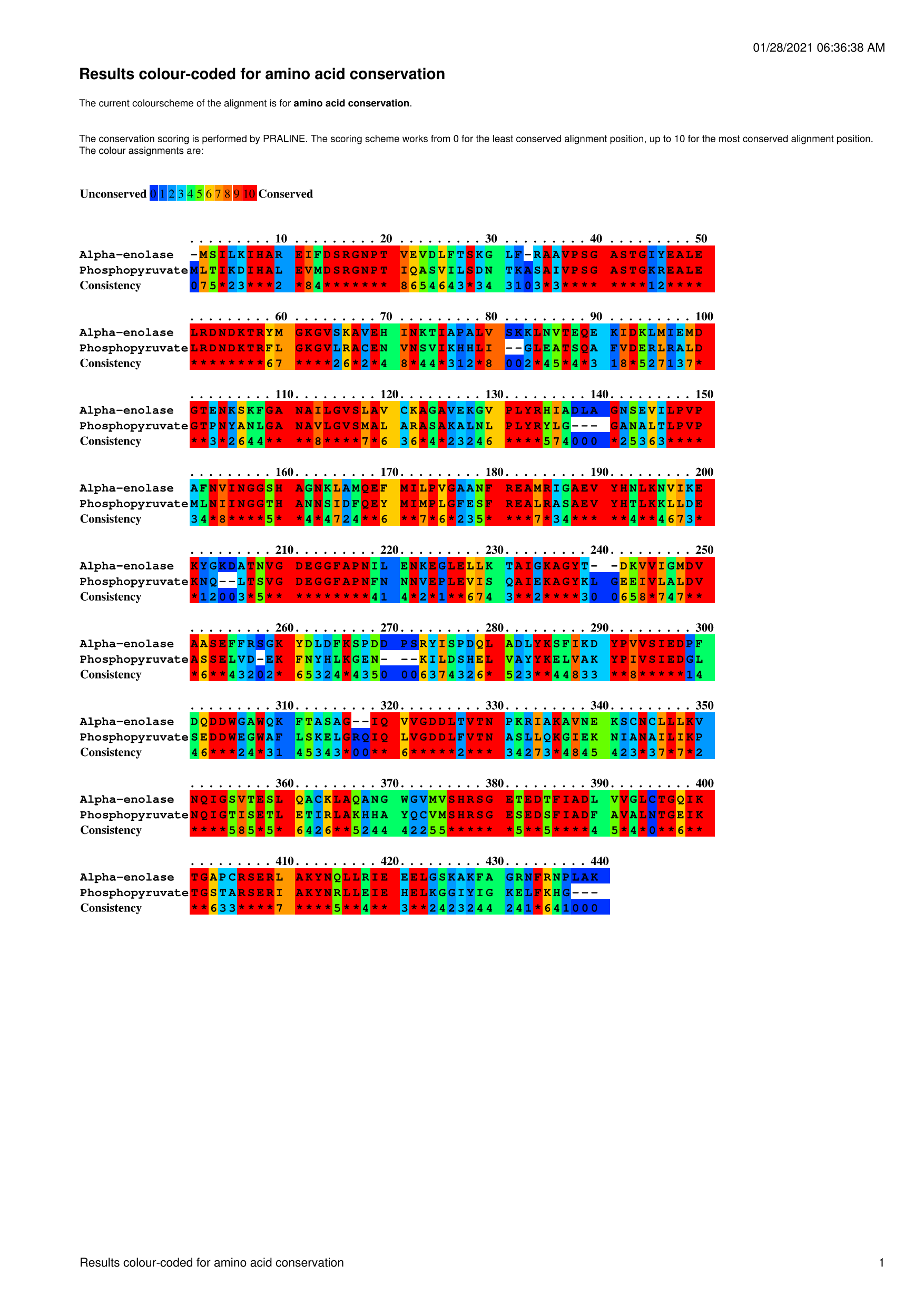

Supplement: S5 Fig — Unconserved sequence are shown with blue color and high conserved sequence with red color. Moderately conserve sequence is showed with green and orange color. (TIFF) [file pone.0281485.s005.tiff]

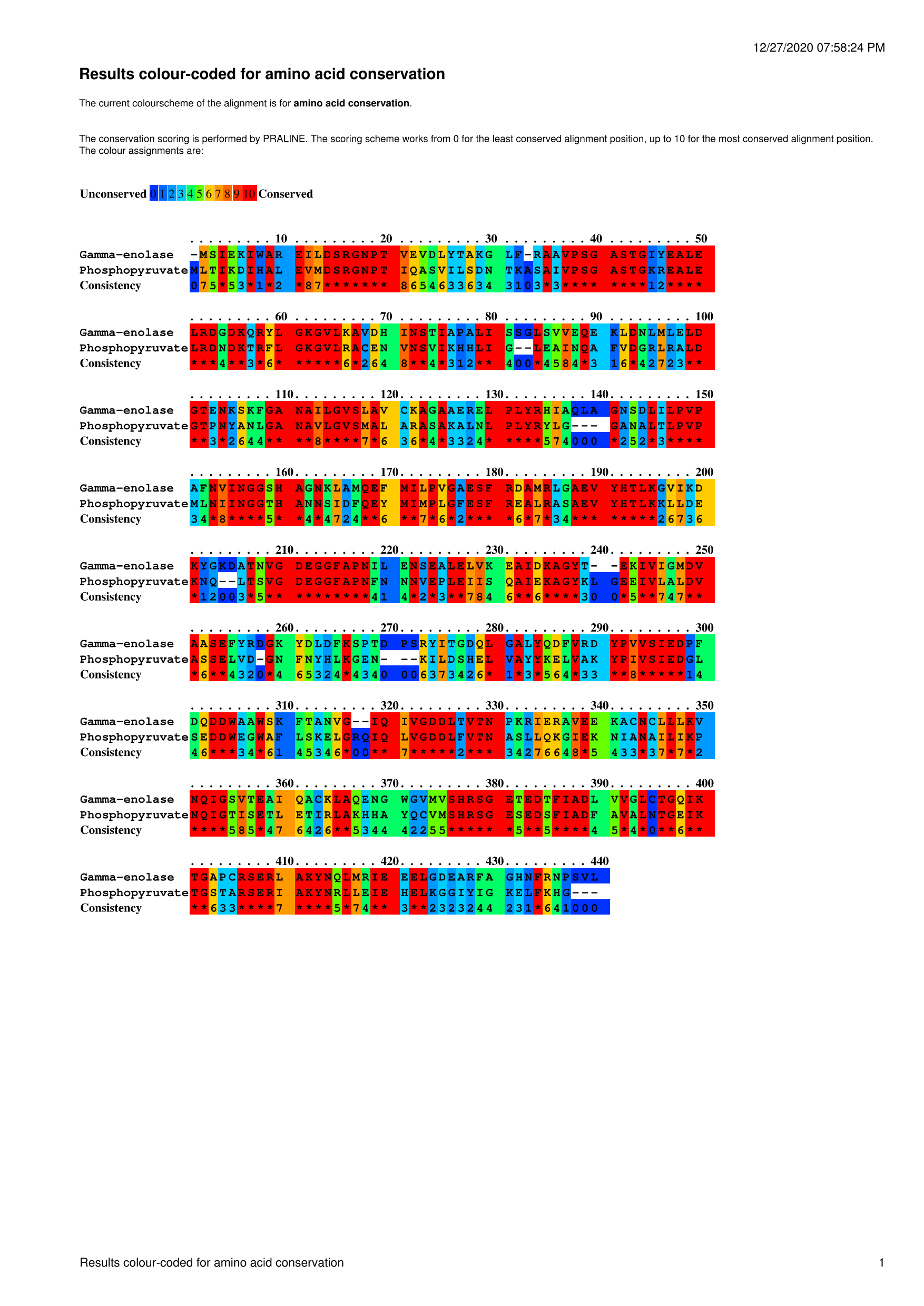

Supplement: S6 Fig — Unconserved sequence are shown with blue color and high conserved sequence with red color. Moderately conserve sequence is showed with green and orange color. (TIFF) [file pone.0281485.s006.tiff]

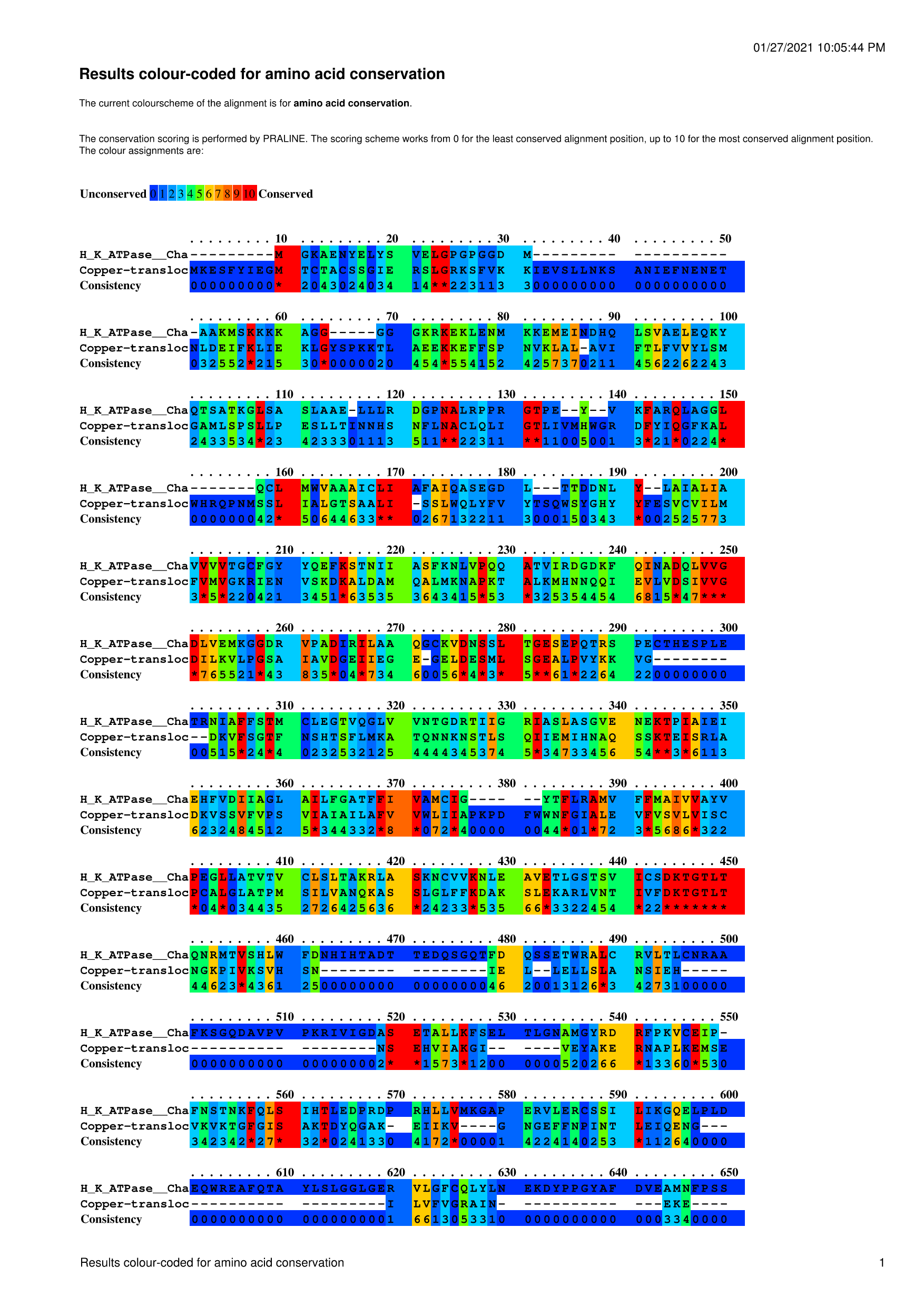

Supplement: S7 Fig — Unconserved sequence are shown with blue color and high conserved sequence with red color. Moderately conserve sequence is showed with green and orange color. (TIFF) [file pone.0281485.s007.tiff]
